# Gastric and Duodenal Ulcer

**Published:** 1907-08

**Authors:** 


### SELECTIONS AND ABSTRACTS.

### GASTRIC AND DUODENAL ULCER.

Mayo divides these ulcers surgically into 2 classes, (1) the indurated ulcer, which can be seen and felt during operation by means of the scar, (2) the nonindurated ulcer, which cannot be indentified from the outside of the stomach or duodenal wall—indeed, it is often difficult to find it even when the stomach is opened. The failures of surgery usually occur in this second group, because the ulcer is not located and its existence may be problematical, or the condition is confounded with pyloric spasm, atonic dilatation, gastroptosis, and the gastric neuroses, or other nonsurgical conditions, or the ulcer does not indicate operation, as it does not interfere mechanically with the progress of food. The value of surgical contribution to our knowledge of nonindurated ulcer is negative, and consists in teaching errors as to diagnosis and pointing out lines of future progress. At the present time the author does not consider that a diagnosis of mucous or other undemonstrated ulcer indicates a surgical operation unless there are such complications as perforation, haemorrhage, or obstruction.—Annals of Surgery, June, 1907.

